# Nature-based solutions could offset coastal squeeze of tidal wetlands from sea-level rise on the U.S. Pacific coast

**DOI:** 10.1038/s41598-025-93437-z

**Published:** 2025-04-03

**Authors:** Karen M. Thorne, Kevin J. Buffington, Michael J. Osland, Bogdan Chivoiu, James B. Grace, Nicholas M. Enwright, Glenn R. Guntenspergen

**Affiliations:** 1 U.S. Geological Survey, Western Ecological Research Center, One Shields Ave., Davis, CA 95616 USA; 2 U.S. Geological Survey, Wetland and Aquatic Research Center, 700 Cajundome Blvd, Lafayette, LA 70506 USA; 3 Contracted to the U.S. Geological Survey, Wetland and Aquatic Research Center, Cherokee Nation System Solutions, Lafayette, LA 70506 USA; 4 U.S. Geological Survey, Eastern Ecological Science Center, Beech Forest Rd, Laurel, MD 20708 USA

**Keywords:** Wetlands ecology, Climate-change ecology

## Abstract

In this study, we explored the opportunities for tidal wetland landward migration in response to sea-level rise on the Pacific Coast of the United States. By employing a systematic spatial approach, we quantified the available space for wetland migration with sea-level rise across 61 estuarine drainage areas. Although many of the existing tidal wetlands are small patches, our analyses show that 63% of the estuaries lacked the landward migration space needed to replace current tidal wetland extent, thereby threatening a wide range of protected species and ecosystem services. Developed lands and steep topography represent common barriers to migration along the Pacific coast, especially in central and southern California. The available wetland migration space consists primarily of agriculture, pasture, and freshwater wetlands, with most of the area available for migration occurring in just a few watersheds. In most watersheds tidal wetland migration would only occur with human intervention or facilitation. The greatest amount of area available for wetland migration was in the San Francisco Bay-Delta and Columbia River estuaries, together accounting for 58% of all available migration space on the Pacific Coast. Nature-based solutions to reduce tidal wetland loss from sea-level rise can include restoration in suitable areas, removal of barriers to tidal wetland migration, and elevation building approaches. Tidal wetland restoration opportunities could increase area by 59%, underscoring it as a plausible approach to prevent tidal wetland loss in those estuaries and a viable Nature-based solution. 54% of estuaries building elevations of existing tidal wetlands may be the most feasible approach needed. Our analyses illustrate the importance of management efforts that use Nature-based approaches to prevent tidal wetland ecosystem and species loss over the coming decades from sea-level rise.

## Introduction

**Fig. 1:**
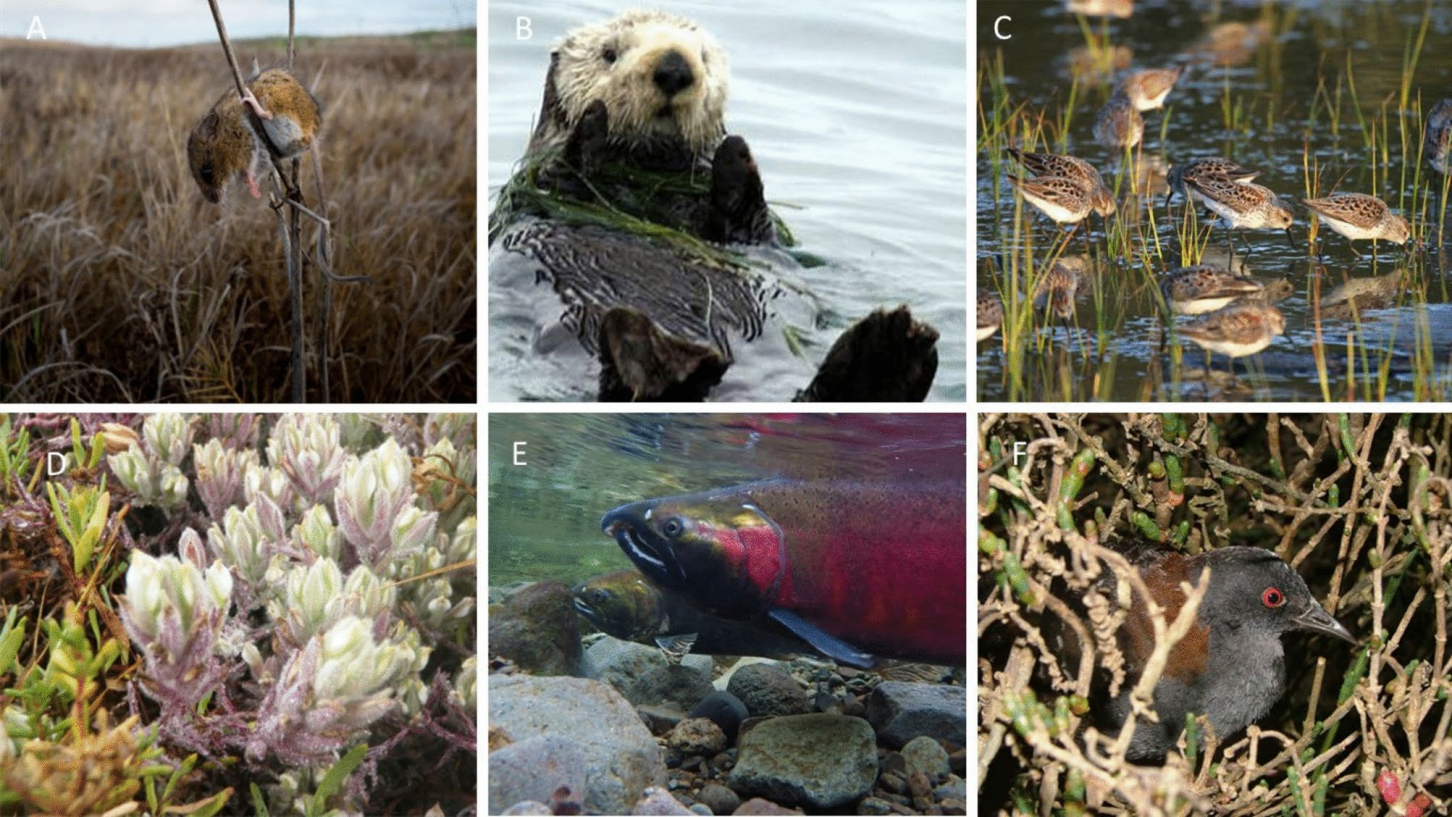
Tidal wetlands support a range of ecosystem services for people and support biodiversity by providing a range of habitats for wildlife and fish. Many species are migratory, endemic, or protected. (**A**) Federally and state endangered salt marsh harvest mouse (*Reithrodontomys raviventris*; photo credit: B Thein, U.S. Fish & Wildlife Service), (**B**) sea otter (*Enhydra lutris,* photo credit: U.S. Fish & Wildlife Service*)* (**C**) Migratory least sandpipers (*Calidris minutilla*; photo credit: D. Ledig, U.S. Fish & Wildlife Service), (**D**) Federally endangered Salt marsh bird’s beak (*Cordylanthus maritimus*; photo credit: L. Cox, U.S. Fish & Wildlife Service), (**E**) Coho salmon (*Oncorhynchus kisutch,* photo credit: Bureau of Land Management), (**F**) California black rail *(Laterallus jamaicensis coturniculus*, photo credit: U.S. Geological Survey). All photos are in the public domain.

A rapidly warming climate poses many challenges to biodiversity, ecosystems, and human communities. Through changes in mean and extreme conditions, and variability^1^, this new climatic environment threatens the maintenance of biodiversity and creates critical challenges for conservation. At the 2022 United Nations Convention on Biological Diversity, a worldwide initiative was adopted to conserve 30% of natural lands and seascapes globally, with the United States (U.S.) committing to protect 30% of their lands and oceans by 2030^2^. In many regions, this can be accomplished through conservation planning and ecosystem restoration, though threats from climate change and sea-level rise make these goals more difficult to achieve, especially along developed coastlines. The world has about 620,000 km of coastlines, with over one-third of the total human population living within 100 km of the coast^3^ and human populations along coasts are projected to continue to increase^4^. Coastlines have a variety of habitats including estuaries that have been recognized as particularly important for their economic, recreational, and cultural value to people (Fig. 1). Changing climate and accelerating sea-level rise threaten estuaries, with increased risk of chronic high tide flooding, flooding during storms and hurricanes, permanent inundation, and ecosystem loss across low-lying and erodible coastlines^5–8^. Rates of sea-level rise are likely to continue accelerating even under the lowest greenhouse gas emission scenarios, with increasing sea-level rise rates more likely under higher emission scenarios and associated rapid melting of ice sheets^9–11^. A recent study suggested that between $4–6 billion USD would be needed to adapt and mitigate for sea-level rise impacts in Los Angeles County alone, which has 120 km of coastline^12^. Our response to climate change will require a science informed decision-making framework to preserve estuaries along the coast of the U.S^13^.

Tidal wetlands cover about 6% of the world’s land surface and occur in sheltered or low-energy estuaries and lagoons^11,14^. Wetlands are under pressure by human activities and development, with over 70% of wetlands lost globally over the last century^15^. There is a total of 2.59 million ha of tidal wetlands in the U.S. primarily with about 97% located along the East Coast and Gulf Coastline^16^, underscoring the need to fully understand the potential cascading effects of sea-level rise for policymakers and managers to implement adaptation strategies. Tidal wetlands provide a range of societal benefits with economic and recreational value being well documented^8,17,18^. In addition, in the U.S. these ecosystems also support a wide range of species at risk, including sea otters (*Enhydra lutris,*^19^), Atlantic salmon (*Salmo salar,*^20^), spoonbill sandpiper (*Calidris pygmaea*^21^), and the California Ridgway’s rail (*Rallus longirostris obsoletus*^22^), which is often not well recognized (Fig. 1). Due to tidal wetland loss, protection and restoration efforts have become more common. Tidal wetland restoration comes in many forms and depends on estuary conditions. One of the most common and effective restoration approaches is the reintroduction of tidal waters by removing dikes or levees, which can return the physical, biological, or chemical characteristics to a natural state^23^. Improving tidal wetland connectivity and rehabilitation by repairing the function of degraded wetlands is also a common practice^23^.

Tidal wetlands can maintain their position within the tidal frame and avoid submergence from rising sea levels by building their surface elevations through biogeomorphic processes including sediment capture and organic contributions^24^. Additionally, tidal wetlands can adapt to rising seas by migrating upslope to colonize suitable elevations^25,26^. Upslope migration is an emerging topic of importance given the urgent need to identify options to facilitate wetland adaptation to sea-level rise^25–28^. However, many of these studies have been conducted along low-lying coastlines with the greatest wetland migration potential, prompting us to conduct a detailed assessment for a region with steep topography, extensive urban development, and presumably more limited migration capacity.

Natural or Nature-based solutions (NbS) refer to a form of adaptation that uses natural features in the form of ecosystem modifications to address societal challenges and provide benefits for both people and biodiversity^29^. NbS can involve a range of approaches such as restoration, protection, or even a semi-natural state (e.g., green seawall). NbS have been identified as a preferred approach to mitigating the impacts from climate change^11^, and tidal wetland restoration is identified as an important NbS management perscription and one that is already employed globally^30^. The potential and limits for NbS for tidal wetlands need to be explored and quantified to understand what is suitable for management^23,31^, especially compared to engineering or grey infrastructure alternatives. These considerations motivated our three related questions: (1) Are there opportunities for NbS to prevent tidal wetland loss? (2) Where are the opportunities for tidal wetland migration and restoration? (3) What landcover types could be converted? We use a regional approach for the Pacific Coast of the conterminous U.S. using spatial data and analyses investigating the opportunity for tidal wetland migration (Table 1). Results of our projections were then analyzed to identify the types of impediments (e.g., urban development) to tidal wetland migration and opportunities for NbS in the form of wetland restoration or vertical adaptation to build sea-level rise resilience. Here, we define wetland restoration as an action that involves efforts to reintroduce physical, chemical, and biological processes to an area suitable for the reintroduction of tidal water to facilitate wetland plant development. For our analyses, restoration potential refers to areas with suitable elevations (up to mean higher high water spring, MHHWS) for wetland development that can be restored by the removal of barriers (e.g., levees, dikes) that are currently impeding tidal waters and emergent plant colonization. This straightforward approach is transferable to other coastlines and provides a framework and the information needed to prioritize conservation and management actions.

**Table 1:** In the context of Nature-based Solutions (NbS) different actions can be taken now or in the future for the Pacific Coast. Here, we use three suggested terms to understand the tradeoffs between tidal wetland migration occurring naturally or investing in human intervention.

Term	Definition	Area available (ha)
Tidal wetland	Current area based on coastal change analysis program (C-CAP) data	106,610
Natural migration	The area available for wetland migration that would occur under sea level rise with no human intervention (no NbS)	40,166
Wetland restoration	The area available currently for tidal wetland restoration that resides within current tidal zone	171,410
Facilitated migration	Wetland migration that could occur under sea-level rise if restoration and removal of barriers took place	45,474

## Results

### Current area

Current tidal wetland area across the 61 estuarine drainage areas (EDAs) along the Pacific Coast of the U.S. totaled 106,610 ha with a mean area of 1,748 ha (Table S1, Figure S1). California had the largest amount of current wetland area with 53%, while Oregon had 30% and Washington had 17% (Table S2). Both the smallest and largest tidal wetland areas were in California, with Ventura EDA being the smallest with 4 ha and San Francisco—San Pablo—Suisun Bay EDA with the greatest tidal wetland amount of 48,248 ha (Table S3, Figure S1). 82% of the EDAs had less than 1,000 ha of existing tidal wetlands (Table S3, Figure S1).

### Natural migration

The area available for natural wetland migration to occur under sea-level rise (MHHSW + 1.5 m) along the Pacific Coast of the U.S. totaled 40,166 ha (Table S1), this scenario considers passive migration with no implementation of NbS. Natural migration could allow 38% of the tidal wetland area to persist under sea-level rise when compared with current area. The Gualala-Salmon EDA had zero area available for natural wetland migration and 13 EDAs had less than 20 ha available for natural migration. Twenty-three EDAs had less then 50% of the original area available for natural migration. 29% of the EDAs could increase their current area with natural marsh migration (Table S3).

### Restoration potential

**Fig. 2:**
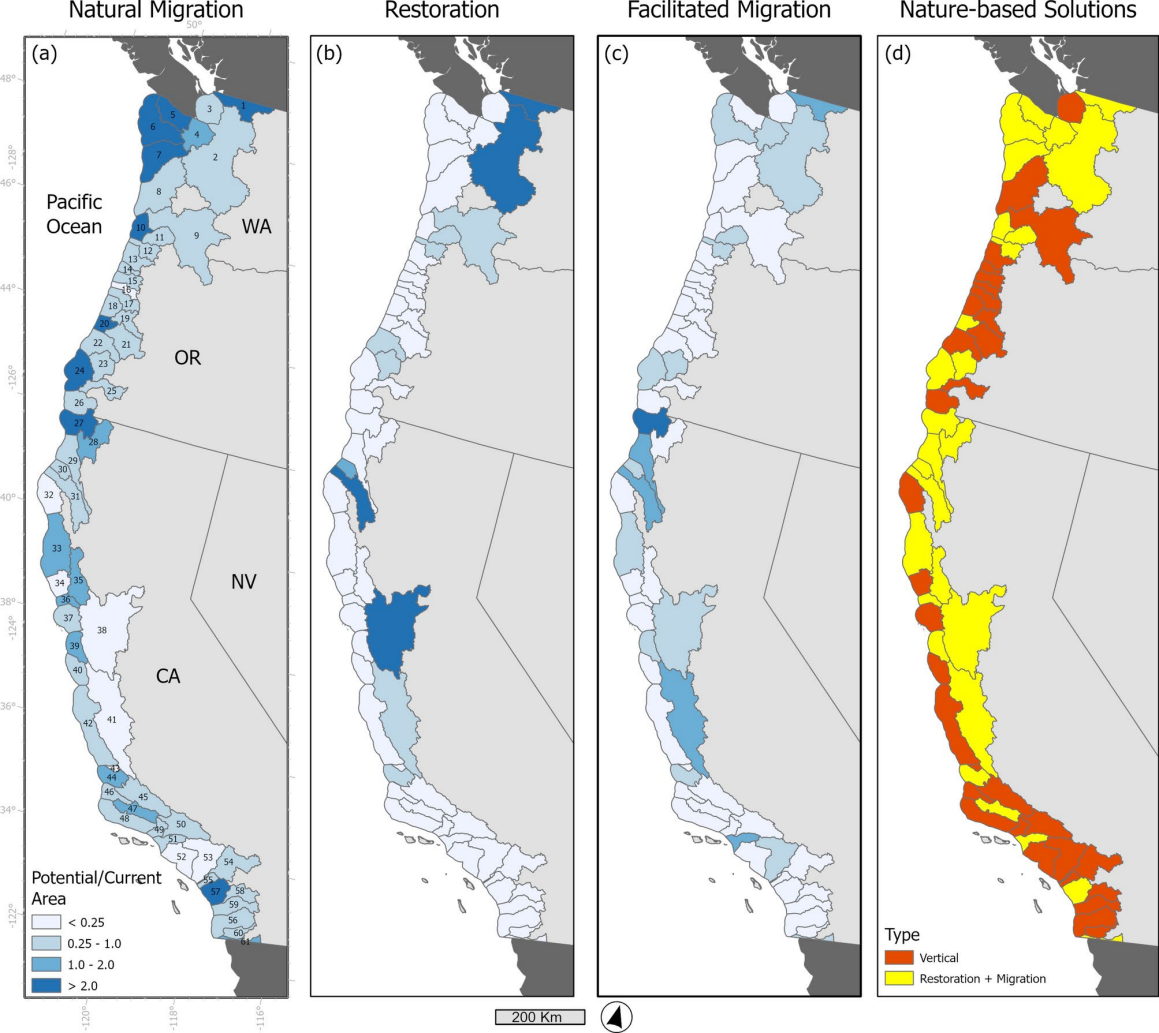
(**A**) Natural migration is how much area is available for wetland migration with no action or intervention by people, numbers represent estuarine drainage areas (EDAs; Dale et al., 2022)  (**B**) The amount available for wetland restoration when compared to current wetland extent by EDAs along the Pacific Coast of the conterminous U.S.. Three EDAs could increase tidal wetland area by over 50%. Refer to Table S2 for EDA names, (**C**) The amount of area available for tidal wetland migration under 1.5 m sea-level rise, illustrating the importance of increasing connectivity between uplands and existing wetland area, (**D**) Dominant type of nature-based solution that would be required to maintain current tidal wetland area across EDAs. Vertical refers to management actions that would help maintain elevations relative to sea-levels, such as thin-layer sediment augmentation.

**Fig. 3:**
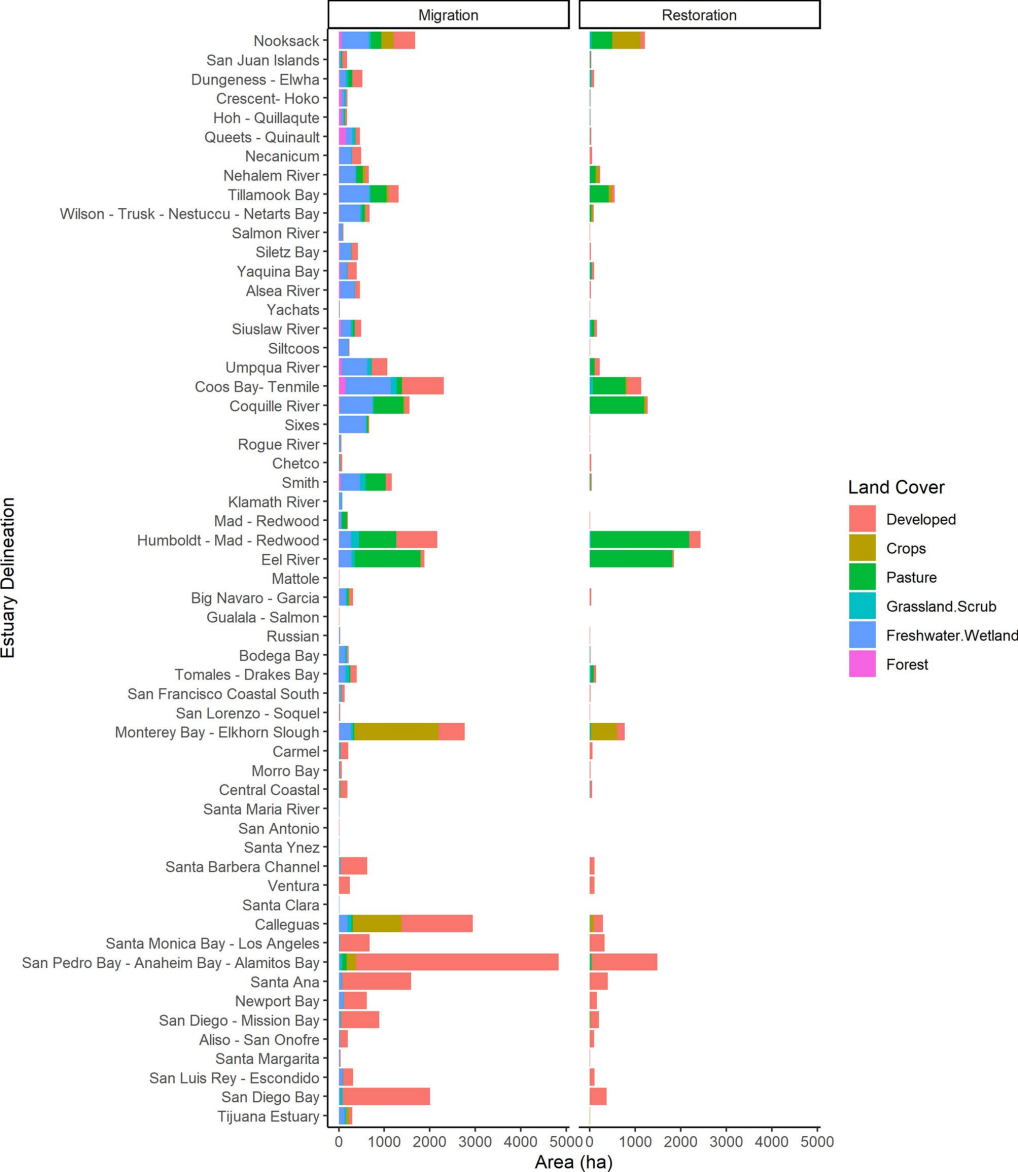
Restoration (right) and wetland migration (left) potential by estuarine drainage areas (EDAs; Dale et al., 2022) across the Pacific Coast of U.S.. Restoration and migration in crop and pasture lands would require active intervention as a NbS. Many wetland migration corridors and restoration opportunities are already developed lands and are considered barriers in this analysis (ArcGIS Pro v3.3.0, ESRI, Redlands, CA, gis mapping software, location intelligence & spatial analytics|Esri). EDAs listed north to south on axis.

**Fig. 4:**
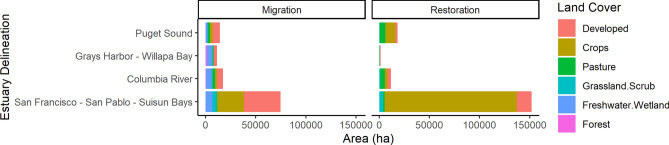
Four estuarine drainage areas (EDAs; Dale et al., 2022) along the Pacific Coast of the conterminous U.S. had most of the area available for wetland migration and restoration. The first two of these EDAs are in Washington, the third EDA straddles the Washington-Oregon border, and the last EDA is in California. The land cover categories indicate the type of lands and ecosystems that would be affected by tidal wetland migration or restoration.

Our analysis shows that the area suitable for restoration is 171,410 ha based on elevation and land cover class (Fig. 2, Figure S3, Table 1, Table S1). Restoration efforts could increase total tidal wetland area by 61% across the Pacific Coast. California had 83% of the area available for wetland restoration, while Washington and Oregon had 10 and 7%, respectively (Table S3). Most of the restoration area was located in the San Francisco—San Pablo—Suisun Bays EDA (80% of the total area) with a possible increase in total tidal wetland area of 284% with 137,185 ha available (Fig. 3, Figure S4, Table S3). Most of this potential area for restoration is currently cropland (Fig. 3, Figure S3). The second largest potential increase in tidal wetland area with restoration was the Puget Sound EDA (16,096 ha, 9% of total), followed by the Columbia River EDA (8392 ha, 5% of total). Four EDAs had the potential to more than double their tidal wetland area with restoration, with Nooksack EDA in Washington having the largest potential with an increase of 3.5 times its current extent. Forty-seven EDAs had minimal potential for tidal wetland area gains with restoration (< 100 ha, Fig. 4). Fourteen EDAs had zero ha available for restoration and 11 EDAs had less than 1 ha available for restoration.

### Facilitated migration

Tidal wetland migration in all EDAs would require active management actions such as the removal of levees and other barriers. With action (removal of barriers to migration, restoration) the potential migration space identified is composed of six land cover types that totaled 157,271 ha across all EDAs (Table S4, Fig. 2). Developed lands were the largest possible area for tidal wetland migration with 71,631 ha (54% of the total) across all EDAs (Fig. 2). However, for this analysis development was considered a barrier for tidal wetland migration. We assumed that human development will be protected from flooding with sea-level rise and therefore will not have the conditions for wetland plant establishment. Therefore, after removing developed lands from the total, only a total of 85,640 ha was estimated as possible migration space for tidal wetlands along the Pacific Coast (Table S4). Two economically important working land cover types identified as possible tidal wetland migration space included crops (34,945 ha) and pasture (10,529 ha). Three coastal ecosystem types were identified as areas for tidal wetland migration—freshwater wetlands (29,239 ha), grassland scrub (8,037 ha), and forests (2,890 ha). Washington and Oregon had the largest amount of freshwater wetland and forests identified as tidal wetland migration space, posing a possible threat to these ecosystem types. The San Francisco—San Pablo—Suisun Bays, Columbia River, and Grays Harbor—Willapa Bay EDAs had the largest area of freshwater wetlands identified as migration space (Table S3, Table S4, Fig. 3). Grays-Harbor—Willapa Bay in Washington had the largest area of forests identified as tidal wetland migration space. Pasture was the dominate landcover type in some EDAs, such as Columbia River EDA, Puget Sound EDA, and Eel River EDA. The San Francisco—San Pablo—Suisun Bays EDA had over 26,755 ha available for tidal wetland migration that are currently crops, while all other EDAs had less than 2,500 ha. San Francisco—San Pablo—Suisun Bays EDA also had the largest amount of grassland/scrub available for tidal wetland migration (Table S3, Fig. 2).

The total tidal wetland migration potential for all EDAs was 85,640 ha with a mean of 1404 ha (SD = 5198) (Fig. 2, Figure S2). The greatest amount of area available for migration was in the San Francisco—San Pablo—Suisun Bay EDA with 38,355 ha, and the second greatest was the Columbia River EDA with 11,437 ha (Fig. 3), together accounting for 58% of all migration space available across all EDAs. Twenty-seven EDAs had less than 100 ha available for migration, with over 50% of the EDAs having less than 200 ha available for migration (Fig. 4). Thirty-eight EDAs (60%) did not have enough migration space to replace existing tidal wetland area (Fig. 4). Twenty-four EDAs (40%) had enough migration space for full replacement, with Smith, Necanicum, and Sixes EDAs (all riverine estuaries) having the potential of increasing tidal wetland area 5–7 times more than their current extent. 46% of the EDAs had total restoration and migration area greater than the current tidal wetland area, we identified these as needing horizontal NbS to facilitate migration (Fig. 4). The remaining 54% of the EDAs would require in-place NbS given there is less opportunity for full area replacement with restoration and migration.

## Discussion

There is considerable uncertainty in predicting future climate and sea levels as they are largely dependent on human behaviors and greenhouse gas emissions over the coming years (IPCC 2022), making it difficult to plan for potential impacts and develop adaptative management prescriptions. Our results illustrate that tidal wetland migration cannot replace existing tidal wetland extent in most places along the Pacific Coast of the U.S. without intervention, and therefore additional management intervention is critically needed to protect ecosystem services and prevent biodiversity loss. This finding for the Pacific Coast of the U.S. contrasts strongly with other analyses for the U.S. and globally. For example, using a meta-analysis, Kirwan et al.^32^ suggested that tidal wetland vulnerability may be overestimated by not considering wetland migration. A global analysis by Schuerch et al.^33^ suggests there could be up to 60% gains in tidal wetlands given migration space and constant sediment supply, and Murray et al.^34^ also showed global gains in some regions over 20 years. Our analysis demonstrates the importance of regional analyses at finer spatial scales and that there may be ‘hot spots’ where loss of tidal wetlands and their associated biodiversity may occur, which is important to consider and prioritize for NbS measures.

### Biodiversity implications

There is great uncertainty on how plants, animals, and other species will adapt to changing environments and if relocation is even possible, which is often called the ‘stationary niche’ concept, versus species being able to shift their realized niches and change within their landscape^35,36^. Forecasts for species under climate change usually define suitability by the current realized niche, but their current niche may only represent a subset of their tolerable conditions^37^. Less explored is the relative importance of these concepts to the conservation concerns related to accelerating sea-level rise and tidal wetland species, which are assumed to have more narrow fundamental niches^38^. The relative importance of different niche variables (i.e., tidal water depth, soil type, competition) may vary among taxa and across space and over time^39^. Exposure-based assessments could provide insight into sensitivity and plasticity of species. A greenhouse experiment demonstrated that Pacific Northwest tidal wetland plants may be more sensitive to changes in salinity than flooding amounts^40^. Whereas Janousek et al.^41^ found overlap in wetland plant species niches across the Pacific Coast illustrating plasticity. In addition, tidal wetland avian species exhibit specialization in foraging ecology and bill morphology^42,43^ demonstrating narrow realized niches.

Our results showed that 62% of the EDAs along the Pacific coast do not have enough migration space to replace current tidal wetland area under a 1.5 m sea-level rise scenario, with 44% of EDAs having less than 100 ha available. These small tidal wetland parcels may not be able to support their current plant and animal communities given their small projected size in our analysis. We hypothesis that species and their ability to cope, adapt, or adjust to changing sea level will be limited for many endemic or obligate species^44^. For example, the salt marsh harvest mouse (*Reithrodontomys raviventris)* is an endemic protected species in San Francisco Bay-Delta, CA (Federal Register 50 CFR 17.11x), which resides within a narrow elevation range of wetland habitat and is prone to being pushed out of their habitat by high water, which can lead to drowning and predation^45,46^. Also, our results illustrate the conservation concern for rare, protected upland plant species such as soft bird’s beak (*Chloropyron molle subsp. molle*) that could be ‘squeezed out’ due to the lack of upland migration space. Soft bird’s beak does occur selectively within the San Francisco Bay-Delta, which was the location with the greatest area available for tidal marsh restoration, presenting an opportunity to prevent species loss. The San Francisco Bay-Delta has extensive, ongoing efforts for tidal wetland restoration over the coming decades^47^ that will benefit tidal wetland species. In contrast, salt marsh bird’s beak (*Chloropyron maritimum ssp. maritimum)* is also a protected rare species that grows in similar habitat as soft bird’s beak but is distributed across southern California estuaries with very little tidal wetland migration or restoration potential due to development or steep topography^48^. In contrast, as wetlands migrate inland, they will replace other habitat types, such as freshwater wetlands, forests, and grasslands/scrub which could impact biodiversity. Along the Atlantic coast of the U.S. ‘ghost forests’ have emerged as indicators of sea-level rise and salt water intrusion^49^ into the upland, but this land conversion also provided an opportunity for invasive species expansion (e.g., *Phragmites* *australis*) which reduced habitat for the saltmarsh sparrow (*Ammodramus caudacutus*).

### Migration potential

Tidal wetlands develop in the presence of favorable conditions in water levels, plant growth, and sufficient sediment supply. The potential for tidal wetland migration to occur across estuaries is dependent on whether the upland habitat is suitable for emergent plant colonization (beyond elevation), which requires saturated hydric soils of particular grain size and appropriate biogeochemistry^50,51^. If soils are not suitable for hydrophytic plant colonization, shifts in dominant plant communities may occur, but more studies are needed. Studies on the physical and biological mechanisms for upland migration of wetlands in New England and Chesapeake Bay (U.S.) concluded that there is a need to advance understanding of how tidal wetland plants can successfully replace upland forests^28,52,53^, and there is debate if the processes are primarily driven by topography, slope, chronic flooding, extreme storm events, or substrate suitability. In contrast, a modeling study showed that slope and extreme water levels interacted to determine the rate of upslope migration^27^. In our analysis, over 2500 ha of the migration space in the Pacific Northwest was dominated by temperate coastal forests, an ecotone habitat which has been shown to be sensitive to saltwater intrusion^54^. Freshwater wetland was also a large proportion of the migration space in Oregon and Washington, which aligns with concerns identified by Osland et al.^26^ and Grieger et al.^55^. For the conterminous U.S., Osland et al*.*^26^ show that two-thirds of the potential space for tidal saline wetland migration is expected to occur at the expense of freshwater wetlands (i.e., freshwater marshes and forests). Tidal saline wetland migration into these freshwater wetlands is a wetland transformation rather than a wetland gain. There is a need for greater understanding on what will occur within freshwater wetlands with saltwater intrusion and the expansion of salt tolerant plants into their space^56^. Measurements and the understanding of the important biogeomorphic feedbacks among vegetation, geomorphology, soil, and migration are needed to understand the role of these components on feasible tidal wetland migration.

### Nature-based solutions

Our results illustrate an important near-term need to develop and implement NbS actions to prevent tidal wetland loss and maintain their ecosystem services with sea-level rise (Fig. 4). Traditional coastal protection strategies include hard engineered infrastructure such as seawalls and weirs^57^. However, there is a growing interest in ‘soft’ or ‘green’ infrastructure as an approach to benefit both tidal wetlands and society^58–61^. The use of NbS are needed to combat sea-level rise impacts on tidal wetland loss for the Pacific coast and has been championed as an important approach to mitigate loss^60,62,63^. This can come in the form of oyster reef building^64^, seagrass restoration^65^, or dune restoration^66^, with tidal wetland restoration as one of the most widespread approaches^23^.

Tidal wetland restoration involves the recovery or recreation of physical, chemical, or biological characteristics associated with these habitats, often requiring the reintroduction of natural hydrologic conditions such as stream connectivity and connection to tidal waters by removing diversions, dikes, and levees. We found that most areas suitable for restoration (e.g., pasture, crops) must have impediments in place to prevent tidal waters from entering that area (Fig. 2, Fig. 4). In these situations, facilitating tidal wetland upland migration would involve the removal of hydrological barriers such as culverts, levees, and old infrastructure. The use of runnels, which are shallow channels, has also been piloted to assess the efficacy to assist restoration efforts in improving wetland drainage^67^. Thus, restoration can come in many forms including a hybrid of green and grey infrastructure, passive restoration in the form of breaking of levees or dikes^68^, or areas designed with sea-level rise enhancement features^69^. For example, horizontal levees are an emerging idea that consists of a hardened structure that has a wide expanse of natural habitat, usually tidal wetlands between the ocean and upland that naturally buffers rising waters and provides provisions for habitat. The San Francisco Bay-Delta had the greatest possibility for wetland restoration accounting for over 80% of the total area along the Pacific coast. Active restoration may be particularly important for this area given the millions of people that reside here and the number of rare and protected plant and animal species^70^.

Restoration methodologies are already in the management toolbox for most areas and therefore expansion of its application could greatly increase resilience. However, a recent debate in the literature has emerged about restoration benefits and if they truly hold climate benefits^23,71,72^. The rate at which tidal wetlands develop in restoration projects is the greatest uncertainty^73^, for example, declining sediment supply in some estuaries may prevent tidal wetland elevations from building enough for vegetation establishment with rising sea levels^74,75^. Many tidal wetland restorations also have goals for carbon sequestration to meet greenhouse gas offset goals^76^, which is dependent on the rate of vegetation development and accretion, and could lead to methane (CH_4_) emissions a more potent warming greenhouse gas^77^. Our analysis presented here shows clear concerns for biodiversity and other ecosystem services with sea-level rise without intervention. Tidal wetland restoration could offset some impacts, but alternative NbS are needed for many estuaries along the Pacific coast. Concerns about the removal of barriers (dikes, levees etc.) which could alter local hydrodynamic processes and result in changes in water velocity, coastal erosion, and flooding are valid and should be evaluated when considering NbS.

Our analysis showed that 46% of the possible migration space is currently developed lands which we treated as a barrier for tidal wetland establishment. Between steep topography and development our results show that there is not enough space for migration to replace existing tidal wetlands without active intervention; therefore, compelling NbS are those that address vertical resilience (build wetland elevations relative to rising seas) and restoration to increase extent and migration potential (Fig. 4). This is in contrast to other U.S. regions that have shown migration as an important process to build sea-level rise resilience and space is available for migration^25,78^. A study focused in southern California found similar results to our analysis and identified that the majority of wetland migration space is currently developed lands, but would have historically been tidal wetlands^79^. Stein et al.^79^ also suggested implementing vertical elevation building strategies where migration space is lacking. In recent years, a vertical resilience approach to raise wetland elevation by applying sediment slurry to a tidal wetland surface or nearby shallows has become widespread, but with mixed results^80–86^. Other accretion enhancement approaches could be tested and could include revegetation to facilitate sediment trapping^87^, nutrient addition to increase productivity^88^, shoreline stabilization to reduce lateral erosion^60^, and improving hydrology to facilitate sediment deposition^7^.

### Social and economic impacts

Tidal wetlands can provide flood protection, but this ecosystem service may be lost with sea-level rise. Our analysis show that there is limited tidal wetland migration space available and therefore the risk of overall loss of these services is possible for some areas^8^. Linhoss et al*.*^89^ estimated a loss in property values in one estuary in Florida, U.S. as $177 million USD from sea-level rise. A national synthesis estimated 1–7 million people are at risk due to sea-level rise with an estimated GDP loss between $70–289 billion USD/year by 2100^90^. A separate analysis for Californi, U.S. a estimated 480,000 people will be impacted (including large number of low-income communities and communities of color) and $100 billion worth of property impacts^91^. In Puget Sound, Washington a study identified that with 1 m of sea-level rise, annual flood extents will increase risk to $206 million USD. NbS could offer management prescriptions that could benefit local communities and economies if tidal wetland extent is maintained.

Recent studies acknowledge that tidal wetland migration could impact rural low-lying working lands (i.e., pasture, crops) and human communities^92^, but could also provide enhanced flood protection for these communities^61,93^. An analysis evaluating impacts to working lands has not been done for the U.S. Pacific Coast, but our results indicate that 53% of tidal wetland migration space is composed of crop and pasture lands. Most estuaries in southern California are dense urban areas including areas of severely disadvantaged human communities^94^, which could have increased flood risks and loss of other ecosystem services. Most tidal wetland migration space in central and southern California was identified as developed illustrating a flooding concern for these coastal communities. Some regions are implementing new laws or regulations to address the concerns for social justice and sea-level rise (e.g.,^95,96^). The implementation of management strategies to promote tidal wetland restoration or migration can evaluate the impacts to human communities and injustices as well. This can be done within a socio-ecological system framework to understand any mismatched goals or unintended consequences^97^.

## Conclusion

The trajectory of sea-level rise seems clear, but the future rate is uncertain (especially after 2050); therefore, identifying long-term management prescriptions along with short-term actions to prevent tidal wetland and associated biodiversity loss is needed^98^. For the case study presented here, we found a general lack of tidal wetland migration space that doesn’t require intensive human intervention and investment, which poses significant concerns for conservation of these ecosystems. If tidal wetlands are lost extensively across the Pacific coast, there will be a need for major engineered infrastructure by coastal human communities^99^. But, if NbS are implemented early enough, ecosystem services and tidal wetland habitats could be maintained over the coming decades including flood protection to developed lands and biodiversity. This work illustrates the importance of conducting studies at the local and regional scale to investigate the nuances of possible change to inform management action.

## Methods

### Study area

We examined the potential landward migration of tidal wetlands along the Pacific Coast of the conterminous United States (i.e., the states of Washington, Oregon, and California). The northern half of the coastline has an oceanic climate with moderate rainfall and mild/cool temperatures, while most of mid to southern portion of the coastline (California) has a Mediterranean climate, which is warmer and dryer. The coastline is a mosaic of offshore rocks, bluffs, beaches, and tidal wetlands. The inland areas of estuaries in this region are composed of forests, grasslands, agricultural croplands, pastures, and urban development. This region has mixed semi-diurnal tides with an average tide range between 2 and 4 m depending on the location. Tidal wetlands tend to start at Mean High Water (MHW) to above Mean Higher High Water (MHHW)^100,101^. In California, emergent halophytic vegetation at higher elevations include *Limonium californicum, Distlichlis littoralis*, and *Frankenia salina*^41^. Tidal wetland platforms are dominated by *Salicornia spp.* and *Distichlis spicata* with *Spartina spp.* dominating lower elevations^100,41^. In the more temperate climates of Oregon and Washington, tidal wetlands are dominated by *Juncus balticus* and *Potentilla anserina* in the high marsh and *Carex lyngbyei* and *Triglochin maritima* in the low marsh areas^101,41^. The Pacific Coast has about 410 estuaries, bays, and sub-estuaries, across 5700 km^2^ of coastline, with three estuaries—Puget Sound, Columbia River Estuary, and the San Francisco Bay Estuary making up 27% of the area^102^. About 85% of tidal wetlands have been lost along the Pacific Coast^103^ due primarily to expansion of human settlements and agriculture activities. Much of the area is densely populated with over 53 million people^104^. Some of the largest coastal cities in the United States are located on the Pacific Coast; for example, Los Angeles (> 4.5 million people), San Francisco Bay estuary (> 9 million people), and Puget Sound estuary (> 4 million people).

### Tidal wetland analyses

#### Existing tidal wetland

We evaluated the total area of tidal wetlands for 61 estuary drainage areas (EDA) that encompass the conterminous U.S. Pacific Coast^26,105^. We used the 2016 NOAA Climate Change Analysis Program land cover classification product (C-CAP,^106^) for land cover classes (cultivated land, pasture/hay, palustrine emergent wetlands, estuarine emergent wetlands, grassland/scrub, forest, and development). We combined all development categories (low, medium, and high intensity development) into one classification, along with forest (deciduous, evergreen, mixed forests). Barren lands were combined with grasslands due to its limited area. Hereafter named—crops, pasture, forest, grassland/shrub, freshwater wetland, and developed. Next a 10 m resolution NOAA sea-level rise digital elevation model (DEM)^107^ was used and augmented with LiDAR-derived, minimum bin DEM where needed to fill in data gaps. Grids of interpolated tidal datums were obtained from NOAA and resampled to 10 m^26^. Relative elevation was calculated by subtracting the local tidal datum elevation from the DEM. The 30 m C-CAP grid was resampled to 10 m to align with the elevation DEM. Current tidal wetland area was defined as the estuarine and palustrine wetland C-CAP classes that were below the local mean higher high water spring (MHHWS), which is the average of the highest level of spring tides.

#### Tidal wetland restoration

In our analysis, restoration areas were identified as an elevation that would be currently suitable for tidal flooding and the establishment of tidal wetland emergent vegetation. Here, we include locations of former or degraded wetland sites that could have barriers removed and tidal water reintroduced to promote the establishment of hydrophytic vegetation. To calculate potential restoration area, we identified non-wetland areas according to C-CAP that were less than the local MHHWS. This elevation was selected since it was the highest possible tidal water level that would be suitable for tidal flooding and emergent vegetation colonization^41^. Land cover classes that were considered as restoration candidates included crops, pasture, and grassland/scrub. Subsided lands below the limit for vegetation establishment were also included, assuming that either natural development or engineered restoration would be possible. The area of developed land that is below MHHWS was also calculated. We then summarized the potential restoration area by EDA and land cover type.

#### Tidal wetland migration

Here, we focused on the potential for tidal wetland migration in EDAs^105^. Our analyses examine the potential effects of a 1.5 m global mean sea-level rise, which for 2100, corresponds to the Intermediate-High greenhouse gas emission scenario^11^. For 2150, a 1.5-m global mean sea level rise falls between the Intermediate-Low and Intermediate scenarios^108^. We accounted for variation in sea-level rise projections by using an interpolated surface across the Pacific Coast. To calculate potential migration area, we extracted land cover data from areas above current MHHWS and below MHHWS + 1.5 m. Isolated pixels in the migration dataset were removed by using the shrink and expand tools in ArcGIS Pro (Esri, Redlands, CA), applying a three-pixel threshold. The C-CAP landcover classes that were considered appropriate for migration (all categories except development) were the same as for the restoration analysis. Potential migration did not require spatial connectivity to a current tidal wetland or restoration; this assumes that either natural recruitment from the seed bank or active planting to facilitate migration would occur. For facilitated migration we then assumed that potential barriers to migration, such as roads or levees, would be engineered (NbS) to facilitate the establishment of tidal wetlands. Here, we identified suitable areas based on elevation, but did not address feasibility which would be an important future analysis.

#### Analysis

EDAs where the sum of restoration and migration area was greater than current tidal wetland area were categorized as needing horizontal intervention (restoration and migration), while EDAs with less than 1:1 replacement of tidal wetland area were categorized as needing vertical intervention (elevation building).

## Supplementary Information

Supplementary Information 1.

Supplementary Information 2.

## Data Availability

All data generated or analyzed during this study are included in this published article and its supplementary information files. The National Land Cover Database through the Coastal Change Analysis Program (C-CAP) is publicly available at C-CAP Regional Land Cover (noaa.gov).
