# Supplementary material for: Nature-based solutions could offset coastal squeeze of tidal wetlands from sea-level rise on the U.S. Pacific coast
Source: Sci Rep. 2025 Apr 3;15:11443. doi: 10.1038/s41598-025-93437-z (PMC11968946; doi:10.1038/s41598-025-93437-z)
Supplement: Supplementary file 1 — Supplementary Information 1. [file 41598_2025_93437_MOESM1_ESM.docx]

**Nature-based solutions could offset coastal squeeze of tidal wetlands from sea-level rise on the U.S. Pacific coast**

Authors: Karen M. Thorne*^1^, Kevin J. Buffington^1^, Michael J. Osland^2^, Bogdan Chivoiu^3^, James B. Grace^2^ Nicholas M. Enwright^2^, Glenn R. Guntenspergen^4^

1. *U.S. Geological Survey, Western Ecological Research Center, One Shields Ave., Davis, CA, 95616, USA
2. U.S. Geological Survey, Wetland and Aquatic Research Center, 700 Cajundome Blvd., Lafayette, LA, 70506, USA
3. Cherokee Nation System Solutions, contracted to the U.S. Geological Survey, Wetland and Aquatic Research Center, Lafayette, LA, 70506, USA
4. U.S. Geological Survey, Eastern Ecological Science Center, Beech Forest Rd, Laurel, MD 20708, USA

*Corresponding author, email [kthorne@usgs.gov](mailto:kthorne@usgs.gov)

**Supplemental Tables**

**Table S1**. Summary statistics for 61 estuary drainage areas (EDAs) along the Pacific coast of the conterminous United States.

|  | **Tidal Wetland Area (ha)** | | | | | |
| --- | --- | --- | --- | --- | --- | --- |
|  | **Sum** | **Mean** | **SD** | **Minimum** | **Maximum** | **Range** |
|  |  |  |  |  |  |  |
| Current | 106,610 | 1,748 | 6,760 | 4 | 48,249 | 48,244 |
| Natural Migration | 40,166 | 658 | 1,901 | 0 | 11,149 | 11,149 |
| Restoration | 171,410 | 2,810 | 17,645 | 0 | 137,186 | 137,186 |
| Facilitated Migration | 45,474 | 745 | 3,549 | 0 | 27,205 | 27,205 |

**Table S2.** Summary of tidal wetland, restoration, and migration area by state along the Pacific coast of the conterminous United States.

|  | | **Tidal wetland area (ha)** | | |
| --- | --- | --- | --- | --- |
|  | **Current** | | **Restoration** | **Migration** |
| Washington | 18,142 | | 17,853 | 17,512 |
| Oregon | 32,383 | | 11,486 | 19,773 |
| California | 56,086 | | 142,071 | 48,354 |
| ***Total*** | 106,610 | | 171,410 | 85,640 |

**Table S3.** Tidal wetland summary by Estuary Drainage Areas (EDAs). EDAs ordered north to south.

| **EDA** | **Current marsh (ha)** | **Restoration (ha)** | **Percent increase in area with restoration** | **Natural Migration (ha)** | **Facilitated Migration (ha)** |
| --- | --- | --- | --- | --- | --- |
| Nooksack | 315.0 | 1113.0 | 353% | 706.6 | 493.8 |
| Puget Sound | 7564.8 | 16096.2 | 213% | 2305.0 | 4718.0 |
| San Juan Islands | 88.7 | 15.3 | 17% | 57.3 | 15.8 |
| Dungeness — Elwha | 172.3 | 32.9 | 19% | 204.8 | 88.2 |
| Crescent — Hoko | 58.4 | 6.2 | 11% | 156.7 | 0.0 |
| Hoh — Quillaqute | 58.7 | 10.7 | 18% | 124.5 | 24.1 |
| Queets — Quinault | 167.3 | 6.4 | 4% | 360.9 | 3.7 |
| Grays Harbor — Willapa Bay | 9716.9 | 572.3 | 6% | 7421.7 | 831.5 |
| Columbia River | 20640.3 | 8391.8 | 41% | 7239.0 | 4198.4 |
| Necanicum | 45.3 | 0.4 | 1% | 289.4 | 0.0 |
| Nehalem River | 577.8 | 204.7 | 35% | 384.4 | 192.0 |
| Tillamook Bay | 1292.6 | 484.7 | 37% | 692.8 | 400.2 |
| Wilson — Trusk — Nestuccu — Netarts Bay | 822.0 | 66.2 | 8% | 504.9 | 80.2 |
| Salmon River | 244.0 | 0.1 | 0% | 81.5 | 0.0 |
| Siletz Bay | 446.6 | 2.4 | 1% | 282.4 | 0.6 |
| Yaquina Bay | 819.3 | 41.8 | 5% | 188.8 | 0.2 |
| Alsea River | 399.1 | 3.6 | 1% | 360.9 | 0.0 |
| Yachats | 14.4 | 0.0 | 0% | 13.6 | 0.0 |
| Siuslaw River | 1094.9 | 102.4 | 9% | 309.0 | 43.7 |
| Siltcoos | 67.8 | 1.4 | 2% | 219.2 | 0.3 |
| Umpqua River | 1407.6 | 113.6 | 8% | 708.4 | 11.2 |
| Coos Bay — Tenmile | 2392.3 | 826.5 | 35% | 1270.9 | 124.1 |
| Coquille River | 1906.0 | 1241.7 | 65% | 768.7 | 677.8 |
| Sixes | 92.2 | 0.1 | 0% | 619.3 | 36.7 |
| Rogue River | 56.8 | 1.5 | 3% | 37.7 | 5.7 |
| Chetco | 63.8 | 3.6 | 6% | 22.2 | 8.9 |
| Smith | 192.9 | 15.5 | 8% | 586.6 | 445.7 |
| Klamath River | 58.7 | 0.1 | 0% | 63.9 | 10.9 |
| Mad — Redwood | 78.6 | 0.2 | 0% | 65.7 | 113.6 |
| Humboldt — Mad — Redwood | 1124.6 | 2183.2 | 194% | 443.3 | 814.8 |
| Eel River | 825.3 | 1825.4 | 221% | 345.4 | 1462.4 |
| Mattole | 48.6 | 0.0 | 0% | 4.0 | 0.0 |
| Big Navaro — Garcia | 165.9 | 1.0 | 1% | 187.3 | 47.6 |
| Gualala — Salmon | 33.7 | 0.0 | 0% | 0.0 | 0.0 |
| Russian | 17.6 | 0.0 | 0% | 20.0 | 0.0 |
| Bodega Bay | 165.2 | 4.2 | 3% | 182.2 | 0.8 |
| Tomales — Drakes Bay | 679.2 | 85.6 | 13% | 224.8 | 29.8 |
| San Francisco — San Pablo — Suisun Bays | 48248.5 | 137186.0 | 284% | 11149.4 | 27205.2 |
| San Francisco Coastal South | 48.9 | 0.0 | 0% | 53.9 | 14.3 |
| San Lorenzo — Soquel | 25.6 | 0.0 | 0% | 14.8 | 0.0 |
| Monterey Bay — Elkhorn Slough | 1395.6 | 601.2 | 43% | 314.4 | 1879.0 |
| Carmel | 78.2 | 0.0 | 0% | 43.5 | 0.1 |
| Morro Bay | 195.7 | 0.2 | 0% | 18.9 | 0.0 |
| Central Coastal | 22.0 | 7.8 | 36% | 32.2 | 19.8 |
| Santa Maria River | 20.8 | 0.0 | 0% | 10.8 | 0.0 |
| San Antonio | 28.2 | 0.0 | 0% | 11.5 | 0.0 |
| Santa Ynez | 12.5 | 0.0 | 0% | 13.6 | 0.0 |
| Santa Barbera Channel | 141.7 | 0.4 | 0% | 41.7 | 1.8 |
| Ventura | 4.2 | 0.0 | 0% | 3.5 | 0.0 |
| Santa Clara | 20.4 | 0.0 | 0% | 12.0 | 0.0 |
| Calleguas | 729.2 | 86.5 | 12% | 275.8 | 1100.6 |
| Santa Monica Bay — Los Angeles | 84.9 | 0.4 | 0% | 17.8 | 0.0 |
| San Pedro Bay — Anaheim Bay — Alamitos Bay | 716.9 | 49.9 | 7% | 77.3 | 297.6 |
| Santa Ana | 100.5 | 0.2 | 0% | 78.2 | 0.7 |
| Newport Bay | 223.7 | 1.2 | 1% | 107.9 | 0.0 |
| San Diego — Mission Bay | 153.6 | 16.7 | 11% | 51.6 | 5.7 |
| Aliso — San Onofre | 11.0 | 0.0 | 0% | 31.4 | 0.0 |
| Santa Margarita | 32.0 | 0.0 | 0% | 18.7 | 0.0 |
| San Luis Rey — Escondido | 148.8 | 0.8 | 1% | 93.4 | 0.0 |
| San Diego Bay | 98.0 | 0.3 | 0% | 79.4 | 0.0 |
| Tijuana Estuary | 154.8 | 4.4 | 3% | 161.0 | 68.3 |

**Table S4.** Migration area (ha) by landcover type across estuarine drainage are (EDAs). Includes both natural and facilitated migration. Developed lands are included to illustrate areas that could have migration but are prohibited by human development. Landcover classifications from NOAA  Coastal Change Analysis Program (C-CAP). EDAs ordered north to south.

|  | **Area available for tidal wetland migration (ha)** | | | | | |
| --- | --- | --- | --- | --- | --- | --- |
| **EDA** | **Freshwater Wetland** | **Forest** | **Pasture** | **Crops** | **Grassland/ Scrub** | **Developed** |
| Nooksack | 595.5 | 66.5 | 228.4 | 265.4 | 44.5 | 481.3 |
| Puget Sound | 1678.2 | 274.8 | 2244 | 2474 | 352.1 | 7267.9 |
| San Juan Islands | 24.2 | 15.8 | 15.8 | 0 | 17.3 | 109.6 |
| Dungeness — Elwha | 135.8 | 21.8 | 87.3 | 0.9 | 47.2 | 225.5 |
| Crescent — Hoko | 51 | 66.2 | 0 | 0 | 39.6 | 30 |
| Hoh — Quillaqute | 47.3 | 56.1 | 13.6 | 10.5 | 21 | 29 |
| Queets — Quinault | 147.9 | 150.9 | 3.7 | 0 | 62.1 | 96.4 |
| Grays Harbor — Willapa Bay | 5144.2 | 1203.9 | 649.9 | 181.6 | 1073.6 | 3173.7 |
| Columbia River | 5966 | 470.5 | 2370 | 1828.4 | 802.5 | 6099.4 |
| Necanicum | 261.3 | 9.2 | 0 | 0 | 19 | 197.3 |
| Nehalem River | 349.1 | 14.1 | 143.9 | 48 | 21.2 | 81.9 |
| Tillamook Bay | 655.5 | 15.4 | 359.4 | 40.8 | 21.9 | 224.7 |
| Wilson — Trusk — Nestuccu — Netarts Bay | 464.6 | 12.2 | 66.2 | 14.1 | 28.2 | 94.6 |
| Salmon River | 73.1 | 4.6 | 0 | 0 | 3.9 | 21.9 |
| Siletz Bay | 254.9 | 22.5 | 0 | 0.6 | 5 | 132.9 |
| Yaquina Bay | 144.7 | 31 | 0.2 | 0 | 13.1 | 205.1 |
| Alsea River | 316 | 31.6 | 0 | 0 | 13.4 | 101 |
| Yachats | 12.2 | 1.3 | 0 | 0 | 0.1 | 9.6 |
| Siuslaw River | 216 | 46.5 | 43.7 | 0 | 46.5 | 134.3 |
| Siltcoos | 204.6 | 5.2 | 0.3 | 0 | 9.5 | 17.8 |
| Umpqua River | 553.3 | 69.3 | 11.2 | 0 | 85.7 | 349.2 |
| Coos Bay — Tenmile | 1000.7 | 141.9 | 116.9 | 7.3 | 128.4 | 911 |
| Coquille River | 713.3 | 23.3 | 651.1 | 26.7 | 32.1 | 107.9 |
| Sixes | 600.6 | 0.4 | 25.9 | 10.8 | 18.4 | 10.9 |
| Rogue River | 32 | 1 | 5.7 | 0 | 4.7 | 10.1 |
| Chetco | 17.9 | 1 | 8.9 | 0 | 3.3 | 42.3 |
| Smith | 428.3 | 38.9 | 441.4 | 4.3 | 119.4 | 127.5 |
| Klamath River | 62.3 | 0.5 | 10.9 | 0 | 1.1 | 2.4 |
| Mad — Redwood | 51.2 | 4.5 | 113.6 | 0 | 10 | 18.9 |
| Humboldt — Mad — Redwood | 255.1 | 16.3 | 814.8 | 0 | 171.9 | 910.6 |
| Eel River | 273.8 | 5.1 | 1450.5 | 11.9 | 66.5 | 69.4 |
| Mattole | 4 | 0 | 0 | 0 | 0 | 4.5 |
| Big Navaro — Garcia | 140.8 | 10.2 | 26.6 | 21 | 36.3 | 75.1 |
| Gualala — Salmon | 0 | 0 | 0 | 0 | 0 | 11.1 |
| Russian | 18.5 | 0.3 | 0 | 0 | 1.2 | 7.2 |
| Bodega Bay | 140.1 | 0.7 | 0 | 0.8 | 41.4 | 37.5 |
| Tomales — Drakes Bay | 136.2 | 6.5 | 28.8 | 1 | 82.1 | 137.3 |
| San Francisco — San Pablo — Suisun Bays | 6866.7 | 29 | 449.9 | 26755.3 | 4253.8 | 36309.5 |
| San Francisco Coastal South | 46.2 | 1.7 | 0 | 14.3 | 6 | 56 |
| San Lorenzo — Soquel | 12.4 | 0.1 | 0 | 0 | 2.4 | 14.4 |
| Monterey Bay — Elkhorn Slough | 260.2 | 9.7 | 22.1 | 1856.9 | 44.5 | 574.5 |
| Carmel | 23.9 | 1 | 0 | 0.1 | 18.5 | 160.6 |
| Morro Bay | 14.1 | 1.2 | 0 | 0 | 3.7 | 47.7 |
| Central Coastal | 30.1 | 1.2 | 0 | 19.8 | 0.9 | 137.1 |
| Santa Maria River | 10.7 | 0 | 0 | 0 | 0.2 | 0 |
| San Antonio | 9.7 | 0.8 | 0 | 0 | 1.1 | 5.3 |
| Santa Ynez | 13.3 | 0 | 0 | 0 | 0.4 | 0.4 |
| Santa Barbera Channel | 36.1 | 0.9 | 1.8 | 0 | 4.8 | 582.1 |
| Ventura | 3.4 | 0 | 0 | 0 | 0.1 | 239.1 |
| Santa Clara | 10.9 | 0 | 0 | 0 | 1.1 | 3.6 |
| Calleguas | 191.7 | 0 | 25.3 | 1075.3 | 84 | 1571.2 |
| Santa Monica Bay — Los Angeles | 15.3 | 0.6 | 0 | 0 | 1.8 | 659.2 |
| San Pedro Bay — Anaheim Bay — Alamitos Bay | 23.4 | 4.1 | 96.1 | 201.5 | 49.7 | 4452.8 |
| Santa Ana | 62.4 | 0 | 0 | 0.7 | 15.7 | 1506.6 |
| Newport Bay | 103.4 | 0.2 | 0 | 0 | 4.4 | 509.2 |
| San Diego — Mission Bay | 42.3 | 0 | 0.8 | 4.9 | 9.3 | 834 |
| Aliso — San Onofre | 30.4 | 0 | 0 | 0 | 1 | 167.1 |
| Santa Margarita | 17.4 | 0 | 0 | 0 | 1.3 | 17.8 |
| San Luis Rey — Escondido | 87.6 | 0.1 | 0 | 0 | 5.8 | 221.7 |
| San Diego Bay | 31.3 | 0 | 0 | 0 | 48.1 | 1924.3 |
| Tijuana Estuary | 126.9 | 0 | 0 | 68.3 | 34.2 | 70.4 |
| **Total** | **29,240.0** | **2,890.6** | **10,528.7** | **34,945.2** | **8,037.0** | **71,631.4** |

**Table S5.** Restoration area (ha) by landcover type across estuarine drainage are (EDAs). Developed lands are included to illustrate areas that could have restoration but are prohibited by human development. Landcover classifications from NOAA C-CAP. EDAs ordered north to south.

|  | **Landcover Type available for Restoration (ha)** | | | |
| --- | --- | --- | --- | --- |
| **EDA** | **Crops** | **Pasture** | **Grassland/Scrub** | **Developed** |
| Nooksack | 619.7 | 448.1 | 45.2 | 100.5 |
| Puget Sound | 9931.7 | 5807.5 | 357.1 | 2308.4 |
| San Juan Islands | 1.5 | 8.0 | 5.8 | 18.0 |
| Dungeness - Elwha | 0.0 | 4.7 | 28.2 | 68.2 |
| Crescent- Hoko | 0.0 | 0.0 | 6.2 | 7.4 |
| Hoh - Quillaqute | 0.5 | 3.2 | 7.0 | 8.5 |
| Queets - Quinault | 0.0 | 0.0 | 6.4 | 25.2 |
| Grays Harbor - Willapa Bay | 9.0 | 230.8 | 332.5 | 792.1 |
| Columbia River | 2718.3 | 4424.6 | 1248.9 | 3204.6 |
| Necanicum | 0.0 | 0.0 | 0.4 | 49.2 |
| Nehalem River | 72.6 | 119.9 | 12.2 | 23.2 |
| Tillamook Bay | 65.8 | 416.4 | 2.5 | 64.6 |
| Wilson - Trusk - Nestuccu - Netarts Bay | 27.8 | 37.9 | 0.5 | 21.2 |
| Salmon River | 0.0 | 0.0 | 0.1 | 11.0 |
| Siletz Bay | 1.8 | 0.0 | 0.6 | 26.4 |
| Yaquina Bay | 0.0 | 25.6 | 16.2 | 57.2 |
| Alsea River | 0.0 | 0.0 | 3.6 | 21.4 |
| Yachats | 0.0 | 0.0 | 0.0 | 7.1 |
| Siuslaw River | 4.3 | 64.0 | 34.1 | 57.9 |
| Siltcoos | 0.0 | 0.0 | 1.4 | 7.7 |
| Umpqua River | 11.1 | 83.7 | 18.8 | 110.5 |
| Coos Bay- Tenmile | 43.3 | 709.4 | 73.8 | 310.5 |
| Coquille River | 49.1 | 1182.9 | 9.6 | 28.5 |
| Sixes | 0.0 | 0.0 | 0.1 | 5.7 |
| Rogue River | 0.0 | 0.0 | 1.5 | 8.4 |
| Chetco | 0.1 | 0.4 | 3.2 | 29.2 |
| Smith | 0.0 | 13.2 | 2.2 | 30.6 |
| Klamath River | 0.0 | 0.0 | 0.1 | 1.4 |
| Mad - Redwood | 0.0 | 0.0 | 0.2 | 12.2 |
| Humboldt - Mad - Redwood | 0.0 | 2157.9 | 25.3 | 254.5 |
| Eel River | 11.8 | 1801.2 | 12.4 | 21.6 |
| Mattole | 0.0 | 0.0 | 0.0 | 2.5 |
| Big Navaro - Garcia | 0.0 | 0.0 | 1.0 | 30.6 |
| Gualala - Salmon | 0.0 | 0.0 | 0.0 | 3.3 |
| Russian | 0.0 | 0.0 | 0.0 | 4.9 |
| Bodega Bay | 0.0 | 0.0 | 4.2 | 11.0 |
| Tomales - Drakes Bay | 0.5 | 56.7 | 28.4 | 51.4 |
| San Francisco - San Pablo - Suisun Bays | 131737.6 | 1304.5 | 4143.9 | 14674.9 |
| San Francisco Coastal South | 0.0 | 0.0 | 0.0 | 15.7 |
| San Lorenzo - Soquel | 0.0 | 0.0 | 0.0 | 6.0 |
| Monterey Bay - Elkhorn Slough | 576.7 | 6.2 | 18.3 | 166.2 |
| Carmel | 0.0 | 0.0 | 0.0 | 64.2 |
| Morro Bay | 0.0 | 0.0 | 0.2 | 13.1 |
| Central Coastal | 0.0 | 0.0 | 7.8 | 40.6 |
| Santa Maria River | 0.0 | 0.0 | 0.0 | 0.0 |
| San Antonio | 0.0 | 0.0 | 0.0 | 0.9 |
| Santa Ynez | 0.0 | 0.0 | 0.0 | 0.1 |
| Santa Barbera Channel | 0.0 | 0.0 | 0.4 | 108.2 |
| Ventura | 0.0 | 0.0 | 0.0 | 102.1 |
| Santa Clara | 0.0 | 0.0 | 0.0 | 0.7 |
| Calleguas | 76.5 | 3.4 | 6.6 | 203.9 |
| Santa Monica Bay - Los Angeles | 0.0 | 0.0 | 0.4 | 322.5 |
| San Pedro Bay - Anaheim Bay - Alamitos Bay | 18.3 | 15.3 | 16.4 | 1439.0 |
| Santa Ana | 0.0 | 0.0 | 0.2 | 400.6 |
| Newport Bay | 0.0 | 0.0 | 1.2 | 160.6 |
| San Diego - Mission Bay | 12.4 | 0.6 | 3.7 | 184.5 |
| Aliso - San Onofre | 0.0 | 0.0 | 0.0 | 95.3 |
| Santa Margarita | 0.0 | 0.0 | 0.0 | 4.1 |
| San Luis Rey - Escondido | 0.0 | 0.0 | 0.8 | 103.7 |
| San Diego Bay | 0.0 | 0.0 | 0.3 | 368.9 |
| Tijuana Estuary | 1.6 | 0.0 | 2.8 | 7.8 |
| **Total** | **145991.8** | **18926.1** | **6492.4** | **26280.2** |
