# Supplementary material for: Nature-based solutions could offset coastal squeeze of tidal wetlands from sea-level rise on the U.S. Pacific coast
Source: Sci Rep. 2025 Apr 3;15:11443. doi: 10.1038/s41598-025-93437-z (PMC11968946; doi:10.1038/s41598-025-93437-z)
Supplement: Supplementary file 2 — Supplementary Information 2. [file 41598_2025_93437_MOESM2_ESM.docx]

**Nature-based solutions could offset coastal squeeze of tidal wetlands from sea-level rise on the U.S. Pacific coast**

**Authors:** Karen M. Thorne*^1^, Kevin J. Buffington^1^, Mike J. Osland^2^, Bogdan Chivoiu^3^, James B. Grace^2^ Nicholas M. Enwright^2^, Glenn R. Guntenspergen^4^

1. *U.S. Geological Survey, Western Ecological Research Center, One Shields Ave. 95616, Davis, CA, 916-502-2996,
2. U.S. Geological Survey, Wetland Aquatic Research Center, 700 Cajundome Blvd., Lafayette, LA, 70506
3. Cherokee Nation System Solutions, contracted to the U.S. Geological Survey, Wetland and Aquatic Research Center, Lafayette, LA, 70506
4. U.S. Geological Survey, Eastern Ecological Science Center, 11649 Leetwon Rd. Kearneysville, WV, 25430

*Corresponding author, email [kthorne@usgs.gov](mailto:kthorne@usgs.gov)

**SUPPLEMENTAL FIGURES**

**
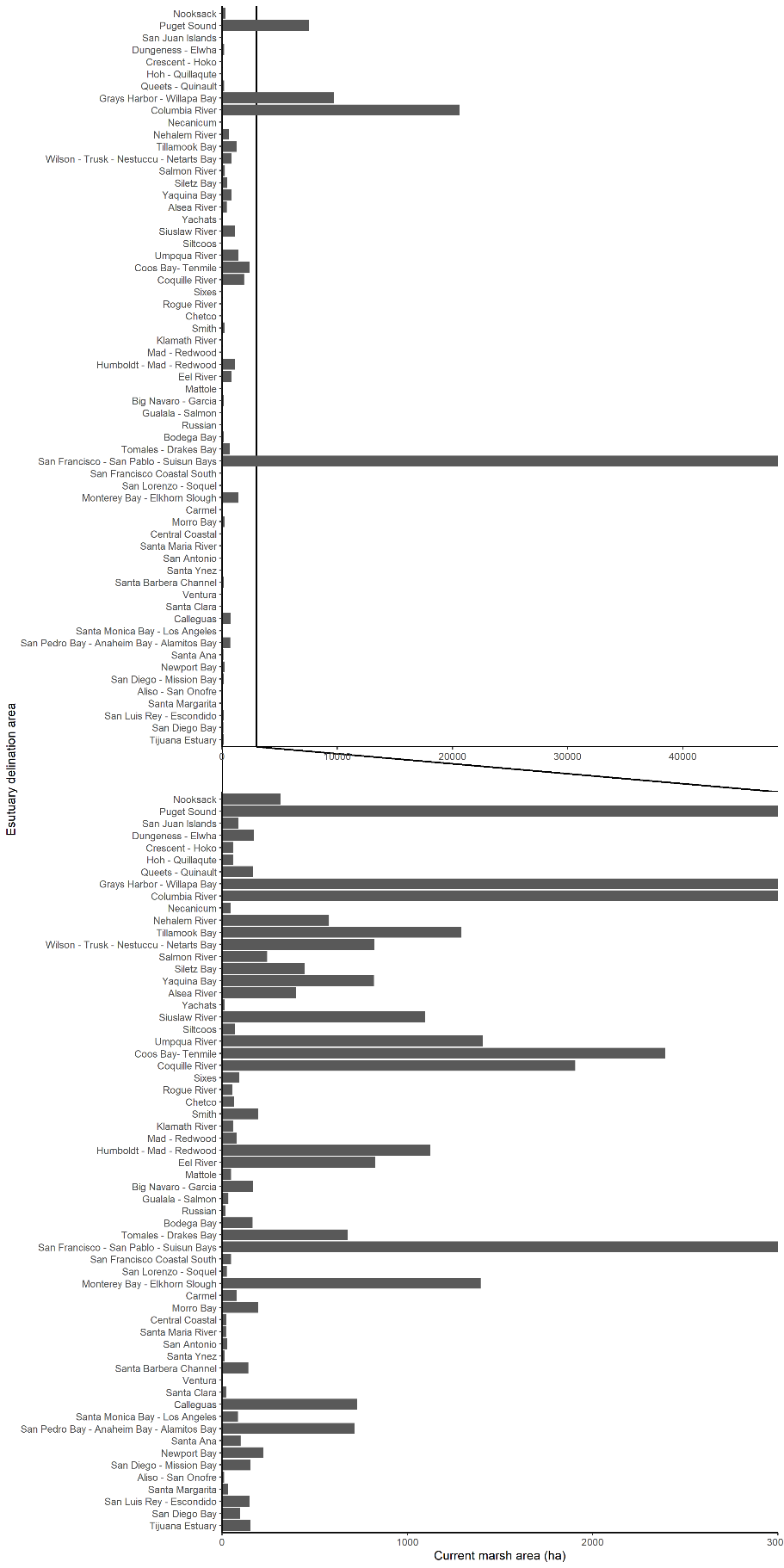
**

**A**

**B**

**Figure S1**. A) Current tidal wetland area by estuarine drainage area (EDA, Dale et al., 2022) along the Pacific coast of the conterminous United States. B) EDA without the top four areas, note change in x-axis.

**
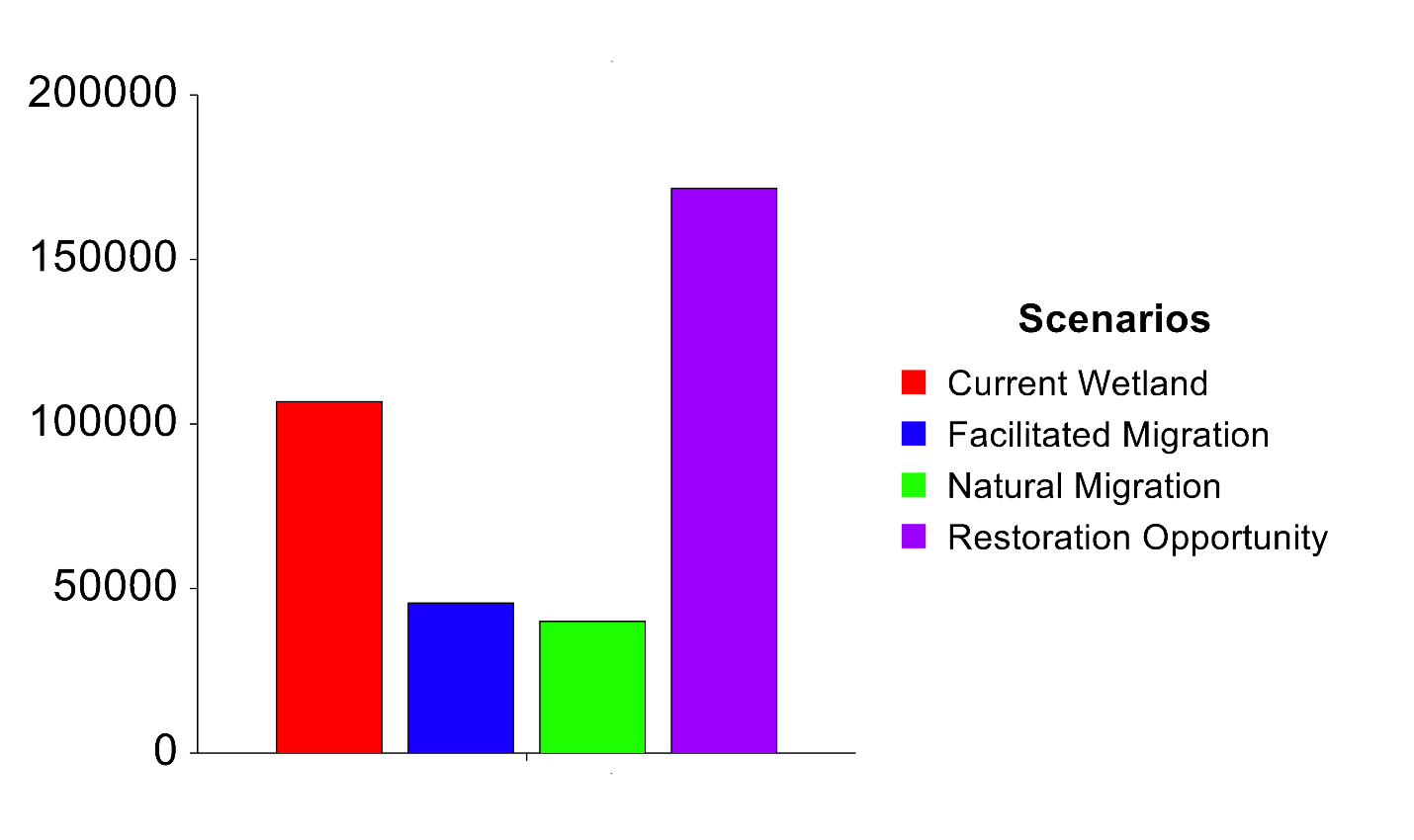
**

Area (ha)

**Figure S2.** Total hectares for current tidal wetland area (estuarine), natural migration, restoration and facilitated migration potential across all estuarine drainage area (EDA, Dale et al., 2022) along the Pacific coast of the conterminous United States.


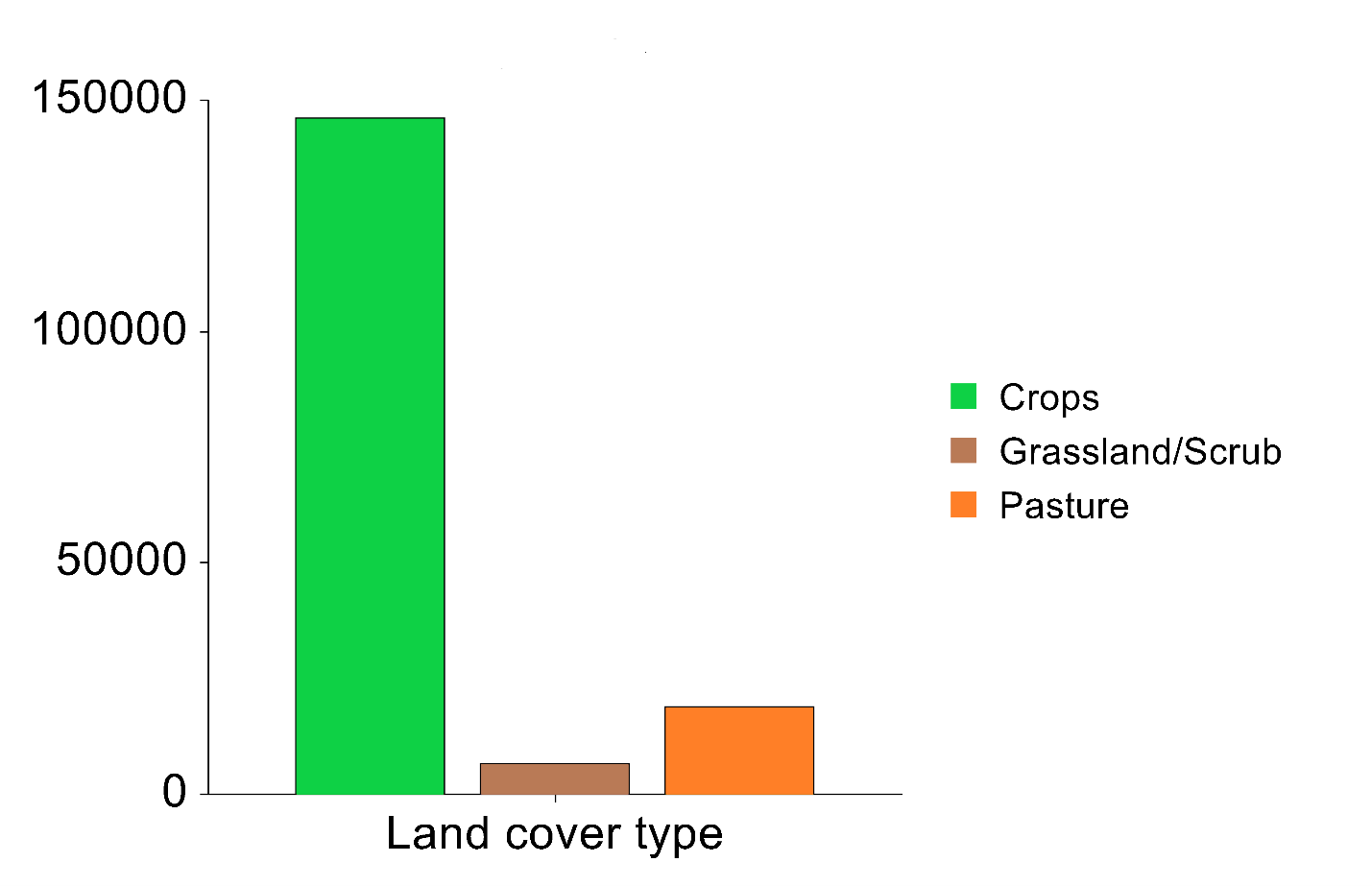


Area (ha)

**Figure S3.** Restoration was identified as an important NbS to increase tidal wetland area to build sea-level rise resilience. Most restoration areas identified are currently in crops with very little opportunities in pasture and grasslands/scrub habitats.

**
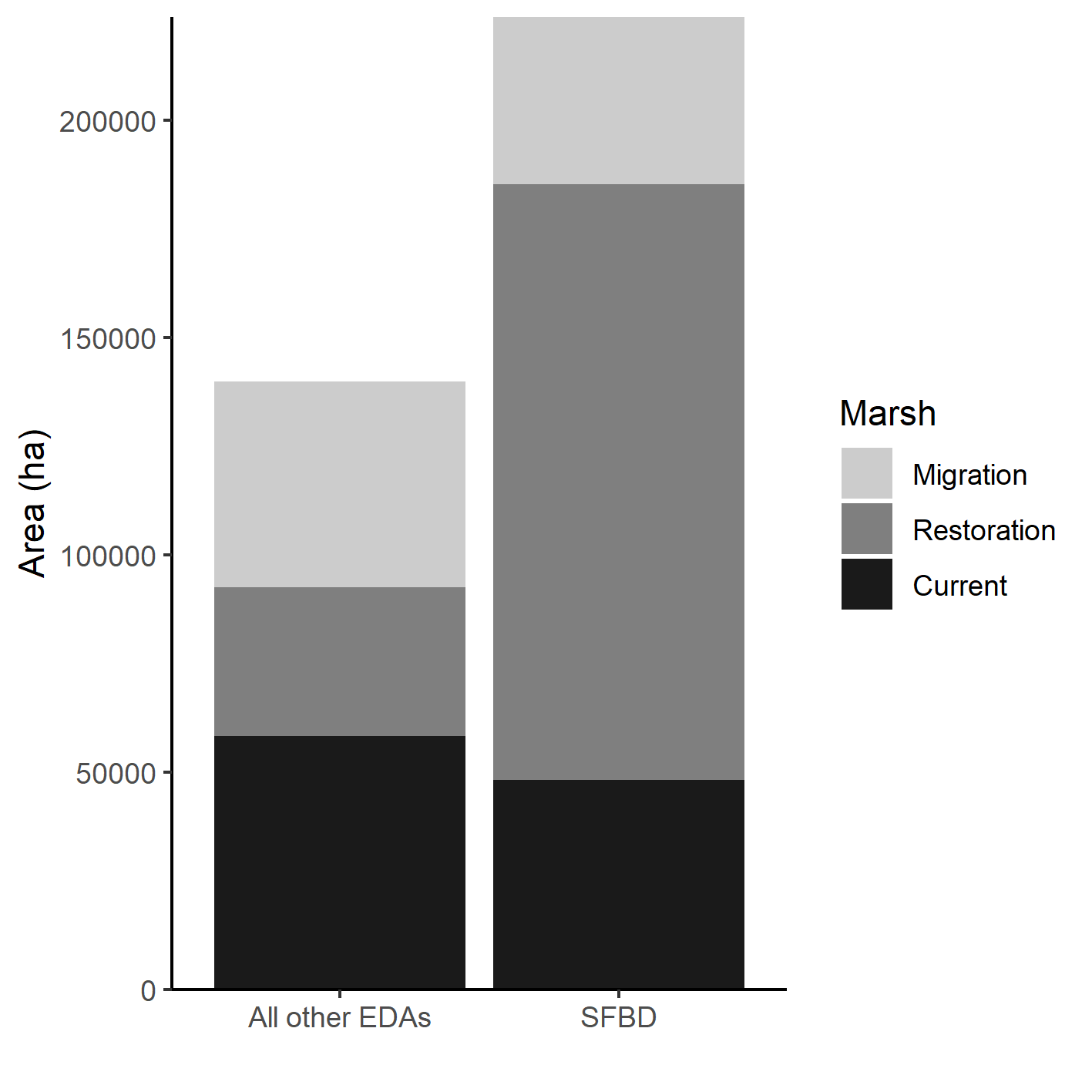
**

**Figure S4**. The comparison of current tidal wetland area, migration potential, and restoration potential between the San Francisco – San Pablo – Suisun Bays (SFBD) estuarine drainage area (Dale et al., 2022) and all other EDAs along the Pacific coast of the conterminous United States. SFBD had the greatest amount of current marsh area, migration potential, and restoration potential when compared to all other EDAs. It also contains most of the Pacific coast area for restoration and migration potential.
